# Differentially expressed seed aging responsive heat shock protein OsHSP18.2 implicates in seed vigor, longevity and improves germination and seedling establishment under abiotic stress

**DOI:** 10.3389/fpls.2015.00713

**Published:** 2015-09-14

**Authors:** Harmeet Kaur, Bhanu P. Petla, Nitin U. Kamble, Ajeet Singh, Venkateswara Rao, Prafull Salvi, Shraboni Ghosh, Manoj Majee

**Affiliations:** National Institute of Plant Genome ResearchNew Delhi, India

**Keywords:** sHSP, chaperone, seed vigor, CDT, stress

## Abstract

Small heat shock proteins (sHSPs) are a diverse group of proteins and are highly abundant in plant species. Although majority of these sHSPs were shown to express specifically in seed, their potential function in seed physiology remains to be fully explored. Our proteomic analysis revealed that OsHSP18.2, a class II cytosolic HSP is an aging responsive protein as its abundance significantly increased after artificial aging in rice seeds. *OsHSP18.2* transcript was found to markedly increase at the late maturation stage being highly abundant in dry seeds and sharply decreased after germination. Our biochemical study clearly demonstrated that OsHSP18.2 forms homooligomeric complex and is dodecameric in nature and functions as a molecular chaperone. OsHSP18.2 displayed chaperone activity as it was effective in preventing thermal inactivation of Citrate Synthase. Further, to analyze the function of this protein in seed physiology, seed specific *Arabidopsis* overexpression lines for *OsHSP18.2* were generated. Our subsequent functional analysis clearly demonstrated that OsHSP18.2 has ability to improve seed vigor and longevity by reducing deleterious ROS accumulation in seeds. In addition, transformed *Arabidopsis* seeds also displayed better performance in germination and cotyledon emergence under adverse conditions. Collectively, our work demonstrates that OsHSP18.2 is an aging responsive protein which functions as a molecular chaperone and possibly protect and stabilize the cellular proteins from irreversible damage particularly during maturation drying, desiccation and aging in seeds by restricting ROS accumulation and thereby improves seed vigor, longevity and seedling establishment.

## Introduction

Seeds, during development acquire remarkable protective mechanisms that allow them to survive desiccation to extremely low water content and to maintain their germinability even after many years of storage. Current evidence indicate that acquisition of desiccation tolerance and seed longevity is a multifunctional trait and diverse mechanisms have been proposed to be involved in acquiring such traits in seeds. As for example, presence and abundance of specific mono, di and oligosaccharides, soluble sugars and specific proteins along with the mechanisms related to ROS removal were shown to play important roles in the acquisition of desiccation tolerance and seed longevity in orthodox seeds (Blackman et al., 1995; Wehmeyer and Vierling, 2000). Previous studies also suggest that orthodox seeds essentially acquire desiccation tolerance during seed development particularly during maturation drying when most of these protective molecules are highly abundant (Roberts, 1973). Among these protective molecules, sHSPs have recently been suggested to play an important role in seed desiccation tolerance and longevity (Wehmeyer and Vierling, 2000; Sun et al., 2002; Tsvetkova et al., 2002; He and Yang, 2013). sHSPs are known to function as molecular chaperones that facilitate protein folding and prevent irreversible protein aggregation during developmental and adverse environmental conditions. Plants synthesize at least 21 different types of sHSPs and are grouped into six different classes on the basis of their subcellular localization and sequence alignments. This sHSP family has members with molecular size ranging from 16 to 42 kDa and invariably contains a conserved ACD in the C-terminal. These sHSPs are believed to play diverse role in plant biology (de Jong et al., 1998). Even though, sHSPs are known more for their extensive role in plant defense against abiotic stresses, their role in seed physiology has recently came into light (Wehmeyer and Vierling, 2000; Sun et al., 2001; Guo et al., 2007; Perez et al., 2009). Certain sHSPs have been reported as preferentially expressed in seeds particularly during development, maturation, and germination, highlighting their role in desiccation tolerance and longevity of seeds (Wehmeyer and Vierling, 2000; Scharf et al., 2001; Sun et al., 2002; Tsvetkova et al., 2002; Neta-Sharir et al., 2005; Volkov et al., 2005; Sarkar et al., 2009; Zhou et al., 2012). A heat shock transcription factor from sunflower (HaHSFA9) overexpressed in tobacco shows an increase in the accumulation of certain sHSPs and consequent improvement of seed vigor and longevity (Prieto-Dapena et al., 2006). Recently, a sHSP from *Nelumbo nucifera* was shown to increase seed germination vigor and seedling thermo tolerance in transgenic *Arabidopsis* (Zhou et al., 2012). In rice, 23 sHSP genes have been identified and majority of these genes are expressed in seed indicating their potential role in seed physiology. However, the role of sHSPs particularly in seed desiccation tolerance and longevity has not been well elucidated (Sarkar et al., 2009).

Rice (*Oryza sativa*) is a monocot model crop which feeds nearly half of the world population. Most of the cultivated rice varieties have a low or null dormancy and variable periods of storage life. Even under best storage conditions, rice seeds lose their viability and germination vigor (He and Yang, 2013). Therefore, a focused effort is required to elucidate and identify the proteins and underlying mechanisms implicated in seed vigor, viability, and longevity. More so, since the production of high quality seeds for food and germplasm preservation is also dependent on seed vigor and longevity. To identify such aging responsive proteins in rice seeds, a proteomic approach has been adopted in this study and eventually a small HSP (OsHSP18.2, a class II heat shock protein, LOC_Os01g08860) has been identified. Subsequently, the *OsHSP18.2* cDNA has been cloned from rice. Further, to get detailed insight of the involvement and participation of this sHSP protein in seed desiccation tolerance and longevity, transcript accumulation was analyzed in different organs, during seed development, aging, germination, and stress conditions. Further, *OsHSP18.2* was bacterially expressed, purified, and biochemically characterized. Finally the ability of this gene to enhance seed vigor and longevity has been analyzed through seed specific overexpression in *Arabidopsis*.

## Materials and Methods

### Plant Materials and Stress Treatments

Rice seeds (*O. sativa* indica cv. PB-1) were imbibed overnight in dark and sown in glass bottles over moist cotton bed and a layer of germination paper. The bottles were then kept for normal growth in growth room 28 ± 1°C and 65% humidity levels. For different stress treatments, 7 day-old seedlings were subjected to various stresses as described by Liu et al. (2009). For time course heat stress, 10 day-old seedlings were transferred to 42°C for 15 min, 30 min, 1, 2, 3, 5, and 24 h.

### Controlled Deterioration Treatment

Seeds harvested on the same day from plants grown under identical conditions were used for all comparisons. Rice and *Arabidopsis* seeds were imbibed for 1 h and then blot dried. The seeds were treated with a combination of high temperature (45°C) and high humidity (100% Relative humidity) for 6 days (for rice) and 4 days (for *Arabidopsis*) to induce artificial aging (Delouche and Baskin, 1973; Verma et al., 2013). CDT treated rice seeds were used for protein extraction for 2 D electrophoresis. Transgenic *Arabidopsis* seeds subjected to CDT were used to score seed viability via germination percentage, tetrazolium assay and H_2_O_2_ accumulation via DAB staining.

### Protein Extraction, 2D Electrophoresis, and Protein Identification

Artificially aged rice seeds (250 mg) were ground to fine powder and homogenized with 2 ml of extraction buffer (20 mM Tris-HCl, pH 7.5, 250 mM sucrose, 10 mM EGTA, 1 mM PMSF, 1 mM DTT, and 1% Triton X-100). Homogenate was centrifuged at 15000 g for 15 min at 4°C and supernatant was collected as total soluble protein. Protein (500 μg) was precipitated with ice cold 100% acetone and pellets were solubilised in 250 μl of DeStreak Rehydration solution (GE) with 2% IPG buffer pH 3-10 NL. The protein sample was used to rehydrate IPG strips 13 cm, pH 3-10NL overnight. Isoelectric focussing (IEF) was performed with rehydrated strips on Ettan IPGphor 3 (GE) at 100V–2 h; 500V–1 h; 1000V–1 h; 8000V–30 min and finally at 8000V–3 h. After IEF, the strips were equilibrated first with 1% (w/v) DTT in equilibration buffer (10 ml of 50 mM Tris-Cl (pH 8.8), 2% SDS, 6 M urea and 30% glycerol) followed by 4% (w/v) iodoacetamide in equilibration buffer. The strips were then loaded on 12.5% polyacrylamide gels for SDS-PAGE. Gels were stained with PlusOne Silver Staining Kit (GE) and analyzed with Image Master Platinum seven software (GE). The protein spots were excised manually and in-gel digestion was performed using trypsin (Sigma). Peptide analysis was carried out on 4800 MALDI-TOF/TOF (Applied Biosystems/MDS SCIEX) and peptides were identified using Mascot search engine run on GPS explorer version 3.6 (Applied Biosystems) using the following parameters: 800–4000 m/z interval MS peak filtering, monoisotopic, MSDB version 20060831 (3239079 sequences; 1079594700 residues), enzyme trypsin with maximum allowance of one missed cleavage, taxonomy Viridiplantae (Green Plants; 247347 sequences), ±100 ppm peptide tolerance, ±0.3 Da fragment mass tolerance, with fixed modification as carbamidomethyl (C) and variable modification as oxidation (M). Protein samples with probability score above default threshold level (*p* < 0.05) as determined by Mascot were considered for further analysis.

### Quantitative Real-time PCR

For real time quantification of the transcripts, total RNA was isolated from rice seeds and seedlings using modified guanidine hydrochloride protocol and TRI reagent, respectively (Singh et al., 2003). Total RNA was treated with DNaseI from Ambion. First-strand cDNA was synthesized from the Dnase treated RNA using the Verso cDNA Synthesis Kit (Thermo). cDNA was then quantified by Nanodrop and dilutions were adjusted for 50 ng/μl. Real time quantification was performed as described in Kaur et al. (2008). Primers used for real time are described in Supplementary Table S1.

### Cloning and Transformation of OsHSP18.2

Full length CDS and 5′ upstream sequence of *OsHSP18.2* (LOC_Os01g08860) was obtained from Ensembl plant *O. sativa* indica group database available at http://ensembl.gramene.org and GenomeIndia’s manually curated database of rice proteins respectively (Gour et al., 2014). Complete ORF was amplified based on the primers designed from these sequences (Supplementary Table S1) from *O. sativa* indica var. PB-1. The coding region was cloned into pJET1.2 vector and sequence was confirmed. Finally the coding region was subcloned into modified pCAMBIA2301 vector under the control of napin promoter for seed specific expression. The construct was then transferred to Agrobacterium strain GV3101 and finally *Arabidopsis* plants were transformed by floral dipping (Clough and Bent, 1998). The 5′ upstream promoter sequence of *OsHSP18.2* was scanned for the presence of *cis*–acting regulatory elements with online available softwares PLACE (Higo et al., 1999) and PlantCARE database (Lescot et al., 2002).

### Bacterial Overexpression and Purification of Recombinant OsHSP18.2

CDS of OsHSP18.2 was subcloned into bacterial expression vector pET23b (Novagen) and transformed into *Escherichia coli* host strain BL21DE3. Transformed cells were grown in LB medium with appropriate antibiotic till A_600_ reached 0.5 and then induced by adding 0.5 mM IPTG. After 6 h growth at 37°C, cells were harvested. Finally, 6X His tagged recombinant HSPs were purified from soluble fraction using nickel-charged affinity columns (GE) following the manufacturer’s protocol.

### Thermal Inactivation Assay

Chaperone activity of OsHSP18.2 was assayed by the method of Lee et al. (1995). CS and purified OsHSP18.2 were combined in equimolar concentration (150 nM) in 50 mM HEPES-KOH buffer, pH-8 with total reaction volume of 500 μl. Controls were made without HSP and with Lysozyme as the control protein. The tubes were incubated in a water bath set at 38°C for 60 min after which the tubes were shifted to 22°C to allow refolding of CS for another 60 min. Twenty micro liter aliquots were removed and CS activity was assayed every 20 min starting at zero minute.

### Citrate Synthase Assay

Citrate synthase activity was measured according to Eigentler et al. (2012). Reaction was set up in a cuvette, 25 μl each of 12.2 mM acetyl coA and 10% triton X-100 was added with 100 μl of 1.01 mM DTNB solution (prepared in 1 M Tris-Cl, pH-8.1). Twenty micro liter of sample was added from the refolding reaction and the final volume was brought to 1 ml. Finally 50 μl of 10 mM Oxaloacetate was added to start the reaction. The absorbance was recorded in a Biorad spectrophotometer set at 412 nm for 2 min at an interval of 20 s. The initial rate of reaction was compared with non-denatured CS and the data was presented as percentage reactivation relative to this activity.

### Tetrazolium Staining

Seeds were stained with 1% solution of 2,3,5-triphenyltetrazolium chloride (Sigma) to differentiate between viable and non-viable seeds according to Verma et al. (2013). Seeds were photographed using Zeiss SteREO Discovery V12 microscope fitted with Axiocam ICc 3 camera.

### DAB Staining

Seeds treated with or without CDT were stained with 3,3′- DAB according to Yi et al. (2010). Seeds were incubated overnight in DAB staining solution (0.1 mg/ml DAB in 50 mM tris actetate buffer, pH 5.0) at 25°C in dark. Next day seeds were bleached in 80% ethanol for 10 min at 70°C. Seeds were photographed using Zeiss SteREO Discovery V12 microscope fitted with Axiocam ICc 3 camera.

### Germination Assay Under Stress Treatments

Seeds from WT, VC, and transgenic lines were harvested at the same time and stored at room temperature till further use. Germination assays were carried out as described in Saxena et al. (2013). Three biological replicates with *n* = 50 seeds were used. Seeds were surface sterilized and plated on to either ½ MS or ½ MS supplemented with NaCl (150 mM), PEG (-0.4 MPa). For heat stress, sterilized seeds were incubated at 45°C for 1 h and then plated on to ½ MS. Seeds were considered germinated when radicle protruded beyond the testa and seedling establishment was considered positive with the emergence of green cotyledonary leaves (Silva-Correia et al., 2014).

### Statistical Analysis

Statistical analysis was performed with one way ANOVA using Duncan’s multiple comparison test to determine significant differences among samples. Differences were taken as significant when *P* < 0.01.

## Results

### OsHSP18.2 is an Aging and Seed Vigor Associated Protein

**FIGURE 1:**
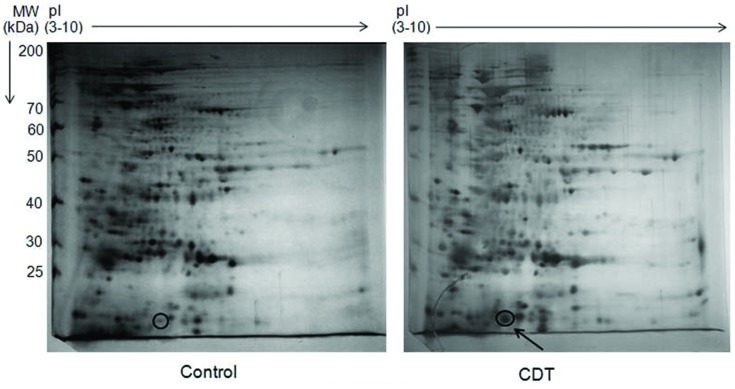
**2D profile of rice seed proteins under control and CDT.** Total 500 μg of protein from Control and CDT treated seeds (6 days) was used for IEF using a non-linear IPG strip of pH 3–10. Second dimension was run on 12.5% SDS-PAGE and gels were stained with silver stain. Gel images were analyzed with Imagemaster 2D Platinum 7.0 (GE Healthcare) and differentially expressed spots were identified. Encircled spot was eluted and sequenced using MALDI-TOF/TOF.

Controlled deterioration treatment has been used widely for quick evaluation of seed vigor, longevity and also to artificially induce aging related changes in seeds (Delouche and Baskin, 1973; TeKrony, 1995; Lanteri et al., 1996; Prieto-Dapena et al., 2006; Oge et al., 2008). Therefore, to identify the seed vigor and aging responsive proteins in rice seed, changes in protein expression after CDT were analyzed using proteomic approach. For CDT, dry seeds were subjected to a combination of high temperature and humidity as described in Section “Materials and Methods.” Subsequently total soluble proteins were extracted from control and deteriorated seeds and were separated by two dimensional polyacrylamide gel electrophoresis (2 D-PAGE). Following silver staining, protein spot showing apparent variation indicated in **Figure 1** was excised from the gel. Excised spot was subjected to trypsin digestion and peptides were extracted and analyzed by MALDI-TOF/TOF. Subsequently peptides were identified using Mascot search engine run on GPS explorer 3.6. Only the best matches with high confidence level were selected. The protein was identified as OsHSP18.2 (LOC_Os01g08860). *OsHSP18.2* is an intronless gene which encodes a protein of 166 aa with an isoelectric point of 5.61 and a predicted molecular mass of 18.2 kDa. Sequence analysis revealed a conserved 90 amino acid alpha crystalline domain, a characteristic feature of sHSP family (Supplementary Figure S1). Pairwise alignment of OsHSP18.2 with its *Arabidopsis* homolog, AtHSP17.0 (AT5G12020.1) shows 59% sequence identity between them (Supplementary Figure S2).

### *OsHSP18.2* Upregulates in Seed, During Seed Maturation, and Upon Accelerated Aging

**FIGURE 2:**
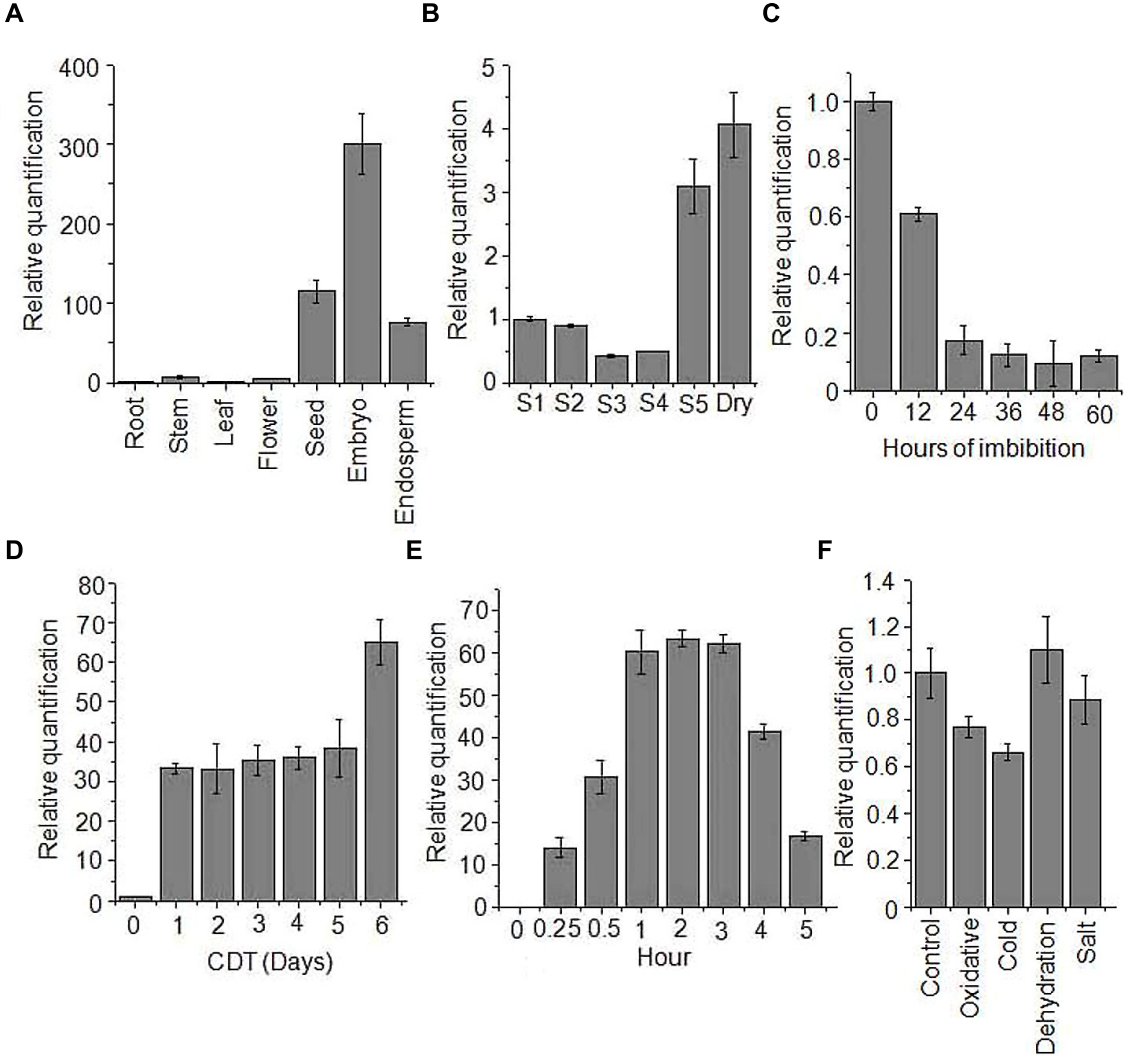
**Expression profiling of *OsHSP18.2*.** Transcript accumulation was analyzed by quantitative real time PCR **(A)** in different organs, **(B)** during seed development, **(C)** during seed germination, **(D)** under CDT, **(E)** under heat stress, and **(F)** under various abiotic stresses. Actin was used as an endogenous control. The relative expression value of each gene was normalized to an endogenous control and calculated using the ΔΔCT method (Applied Biosystems). Expression values are the mean ± SD of three independent biological measurements.

To elucidate the function and mechanism of *OsHSP18.2* in seed maturation and desiccation tolerance, we initially investigated the transcript accumulation of *OsHSP18.2* in seed along with other organs. As shown in **Figure 2A**, *OsHSP18.2* transcript was highly abundant in seed and more specifically in embryo than endosperm. Subsequently, to get the refined view of transcript accumulation during seed development, flowers were tagged according to the day after pollination (DAP) as described by Agarwal et al. (2011). Accumulation of *OsHSP18.2* transcript was found to be relatively low during initial stages of development, i.e., S1 till S4 stage (0–20 DAP) then strikingly increased in the late maturation stage S5 (21–29 DAP) and reached highest levels in dry mature seed (**Figure 2B**). This data essentially revealed that *OsHSP18.2* markedly increased at the later stages of seed development consistent with the time when seed actually acquires desiccation tolerance and longevity.

Transcript accumulation was also studied during germination and data showed a sharp decline in transcript level as germination proceeds (**Figure 2C**). Next, we investigated the correlation of transcript accumulation of OsHSP18.2 and aging in rice seeds. For this, rice seeds were subjected to CDT treatment for 6 days and transcript accumulation pattern was studied. Results clearly revealed a significant increase in transcript accumulation due to artificial aging even after 1 day of CDT which was further increased to more than 60-fold after 6 days of CDT (**Figure 2D**). To examine the possible involvement of *OsHSP18.2* in abiotic stress tolerance, transcript accumulation was checked in rice seedlings challenged with various stresses (**Figures 2E,F**). As shown in **Figure 2E**, heat stress triggered maximum induction of *OsHSP18.2* transcript as expected while other abiotic stresses had minor changes in transcript levels (**Figure 2F**). This analysis clearly revealed that *OsHSP18.2* is upregulated during seed maturation and upon aging and indicates its participation in maturation drying and seed longevity. Transcriptional induction of *OsHSP18.2* in rice seedlings challenged with heat stress also indicates its participation in thermal stress tolerance. Investigation of the 5′ upstream sequence of OsHSP18.2 revealed the presence of many interesting *cis*-acting regulatory elements which are possibly responsible for abiotic stress responsiveness and seed and embryo specific expression. A list of some of these *cis*-acting elements is shown in **Table 1**.

**Table 1:** Summary of key *cis* regulatory elements in the 5′-upstream sequence of *OsHSP18.2*.

S. No.	*Cis* regulatory element	Position	Sequence	Description	Reference
1	2SSEEDPROTBANAPA	-325(+)	CAAACAC	Seed specific expression element	Stalberg et al., 1996
2	CANBNNAPA	-325 (+)	CNAACAC	Embryo and endosperm-specific Expression element	Ellerstrom et al., 1996
3	CBFHV	-89 (+)	RYCGAC	Dehydration responsive element	Xue, 2002
4	DPBFCOREDCDC3	-322, -333, -1461 (+)	ACACNNG	ABA and embryo specific element	Kim et al., 1997
5	DRE2COREZMRAB17	-89 (+)	ACCGAC	ABA and drought responsive element	Busk et al., 1997; Kizis and Pages, 2002
6	EBOXBNNAPA	-236, -308, -332, -723, -1460, -1715, -1842 (+)	CANNTG	Seed specific expression element	Stalberg et al., 1996
7	HSE	-422 (+)	GAANNTTCNNGAA	Heat stress responsive element	Amin et al., 1988
8	LTRE1HVBLT49	-247 (+)	CCGAAA	Low-temperature-responsive element	Dunn et al., 1998
9	LTRECOREATCOR15	-88 (+)	CCGAC	Low temperature responsive element	Baker et al., 1994; Jiang et al., 1996
10	MYBCORE	-551, -1074 (+)	CNGTTR	Dehydration responsive element	Urao et al., 1993
11	MYCCONSENSUSAT	-236, -308, -332, -724, -1460, -1715, -1842 (+)	CANNTG	Dehydration and cold responsive element	Abe et al., 2003; Chinnusamy et al., 2003
12	NAPINMOTIFBN	-1001 (+)	TACACAT	Seed specific expression element	Ericson et al., 1991
13	POLLEN1LELAT52	-64, -353, -640, -917, -1281, -1297, -1414, -1434, -1562, -1934, -1938 (+)	AGAAA	Pollen specific expression element	Bate and Twell, 1998; Filichkin et al., 2004
14	SEF4MOTIFGM7S	-648, -1091, -1652 (+)	RTTTTTR	Seed specific expression element	Allen et al., 1989
15	UPRMOTIFIAT	-124 (+)	CCACGTCA	Unfolded protein response	Martinez and Chrispeels, 2003; Oh et al., 2003
16	UPRMOTIFIIAT	-138 (+)	CCNNNNNNNNNNNNCCACG	Unfolded protein response	Martinez and Chrispeels, 2003; Oh et al., 2003

### Purified Recombinant OsHSP18.2 Forms Higher Order Oligomers

**FIGURE 3:**
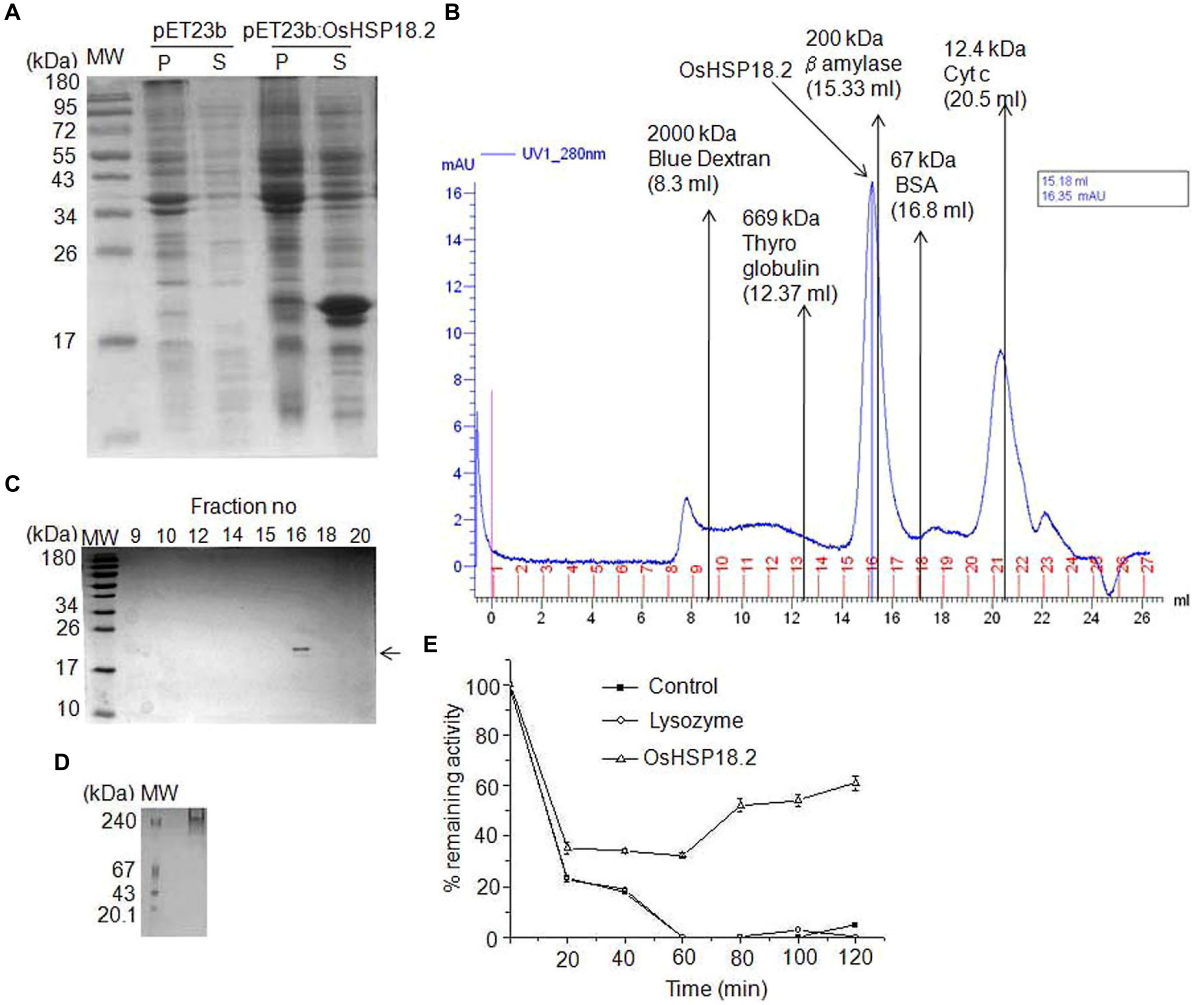
**Characterization of recombinant OsHSP18.2. (A)** Bacterial overexpression of OsHSP18.2 in *Escherichia coli*. Proteins were separated on a 15% SDS-PAGE gel. pET23b empty vector transformed cells were used as a control. Pellet (P) and Supernatant (S) were run for control and *OsHSP18.2* transformed cells along with the molecular weight marker. **(B)** Size exclusion chromatography on Superose 6 column revealed the homooligomeric nature of OsHSP18.2. Purified protein and appropriate molecular weight markers were run and their elution time (V_e_) to void volume (V_o_) ratio (V_e_/V_o_) was plotted against their molecular weight in log scale and fitted in an exponential function where *y* = 512304e^-4.306x^. Approximate molecular weight of OsHSP18.2 was calculated from the above equation as ∼202 kDa which reflects dodecameric conformation. **(C)** Gel filtration fractions as run on 15% SDS-PAGE show a single band of OsHSP18.2 in fraction number 16 corresponding to the gel filtration peak. **(D)** Five percent native polyacrylamide gel shows a protein band of OsHSP18.2 across the 240 kDa marker band. **(E)** OsHSP18.2 prevents irreversible thermal inactivation of CS at 38°C. CS was incubated without HSP (—■—), with lysozyme (—○—) and with OsHSP18.2 (—Δ—) at 38°C for 60 min and then shifted to 22°C for refolding. CS enzyme activity was assayed at the mentioned time points. Values are the mean ± SE of three independent experiments.

sHSPs are known to form multimers with varying number of subunits, most commonly between 2 and 48 subunits (Basha et al., 2012). In order to explore this, we isolated and cloned OsHSP18.2 coding region into bacterial expression vector pET23b and transformed into *E. coli* expression host BL21DE3. The recombinant protein was induced by IPTG and was found to be expressed in soluble phase (**Figure 3A**). Subsequently, 6X His tagged OsHSP18.2 protein was purified using nickel charged affinity chromatography. Size exclusion chromatography was used to check the oligomeric state of OsHSP18.2 and the purified protein was shown to elute as an oligomer of ∼202 kDa thus having approximately 12 subunits which agrees to the dodecameric nature of wheat sHSP16.9 (van Montfort et al., 2001; **Figure 3B**). The gel exclusion fractions were run on SDS-PAGE and revealed the presence of a single band in the 202 kDa peak fraction corresponding to the single subunit size of 18.2 kDa (**Figure 3C**). In order to confirm this oligomeric association of OsHSP18.2, the purified protein was run on native PAGE (**Figure 3D**) which revealed a band across 240 kDa marker band thus endorsing the gel filtration results.

### OsHSP18.2 is an Active Chaperone and Prevents Thermal Denaturation of CS

Many small HSPs are known to have *in vitro* chaperone activity. Therefore, to assess the chaperone activity of OsHSP18.2, we used CS as a substrate for refolding experiments (Collada et al., 1997; Lee et al., 1997). CS monomers (150 nM) were incubated with or without HSP and with lysozyme as control at 38°C for 60 min and a quick decrease in CS activity was observed (**Figure 3E**). Control without HSP and with lysozyme showed 20% remaining activity after just 20 min of heat stress which came down to almost zero after 60 min of heat stress whereas CS with OsHSP18.2 retained 30% activity even after 60 min of heat. After 60 min, all the combinations were shifted to 22°C, temperature permissive for refolding. Even at 22°C, control lacking HSP and lysozyme control did not display any regain in CS activity but CS with OsHSP18.2 showed a regain of 60% of native CS activity. This result clearly demonstrated that OsHSP18.2 functions as a molecular chaperone that facilitates protein folding and prevents thermal denaturation.

### Seed Specific Overexpression of *OsHSP18.2* in *Arabidopsis thaliana* Improves Seed Vigor and Longevity

**FIGURE 4:**
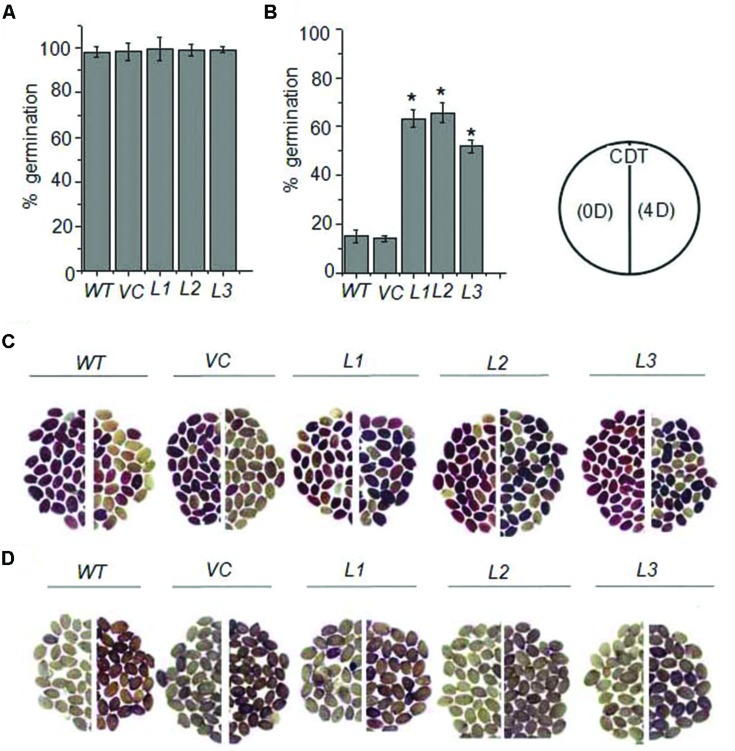
**Germination and viability of *OsHSP18.2* overexpressing seeds after CDT.** Six week-old seeds were subjected to 4 days of CDT as described in Materials and Methods. Germination analysis of WT, empty vector VC, and three *OsHSP18.2* transgenic lines (L1, L2, and L3) before **(A)** and after **(B)** 4 days of CDT. **(C)** Tetrazolium assay for seed viability and **(D)** DAB staining for H_2_O_2_ production of WT, empty vector (VC), and three *OsHSP18.2* transgenic lines (L1, L2, and L3) before (left) and after 4 days of CDT (right). Age matched seeds were used for all germination experiments. In graph the values are mean ± SE of three independent sets (*n* = 50). Asterisk indicate significant difference between WT and test genotypes (^∗^*P* < 0.01).

To examine the functional implication of sHSP in seed vigor and longevity, *OsHSP18.2* was overexpressed in seeds in *Arabidopsis thaliana* using the napin promoter. Seed specific *OsHSP18.2* transcript accumulation was examined through quantitative RT PCR and significant levels of transcripts were observed in seeds of transgenic lines. Based on this analysis, three independent homozygous T3 lines were selected and subsequently used to assess their germination vigor and longevity. To evaluate seed vigor and longevity, seeds were subjected to CDT and germination performance was analyzed. Under normal conditions, transformed and control seeds (empty vector transformed or WT) exhibited 100% germination (**Figure 4A**), however, after CDT, control seeds showed <20% germination as opposed to *OsHSP18.2* transformed seeds where remarkably >50% germination was observed in each line (**Figure 4B**). In addition, TZ staining was carried out to examine the potential viability of these seeds (Berridge et al., 1996). TZ precipitates to red colored 2,3,5 triphenyl formazan by the activity of dehydrogenases present in the living cells thus staining them red. As expected under normal condition, *OsHSP18.2* expressing and control seeds were stained dark red indicating their viability while after CDT, only *OsHSP18.2* transformed seeds exhibited dark red staining (i.e., viable) in contrast to control seeds which remain unstained or were stained pale red (non-viable; **Figure 4C**). Aging in seeds is also accompanied with ROS accumulation (Bailly, 2004). ROS adversely affects cellular proteins and enzymes and renders them inactive. Therefore, we wanted to check if the overexpression of *OsHSP18.2* protects the seed against ROS mediated damage. DAB staining was performed for this purpose as it stains the areas of H_2_O_2_ production (Mao and Sun, 2015). Results clearly demonstrated that WT and VC seeds accumulated more H_2_O_2_ after CDT in comparison to *OsHSP18.2* overexpressing lines (**Figure 4D**). These data clearly indicate that WT or empty vector transformed seeds subjected to controlled deterioration exhibited higher mortality, reduced germination and more H_2_O_2_ accumulation while seeds from transgenic lines overexpressing *OsHSP18.2* exhibited less mortality, better germination and less H_2_O_2_, thus demonstrating the role of *OsHSP18.2* toward maintaining seed viability and longevity during aging.

### *OsHSP18.2* Transformed *Arabidopsis* Seeds Display Improved Seed Germination and Seedling Establishment Under Abiotic Stress Conditions

**FIGURE 5:**
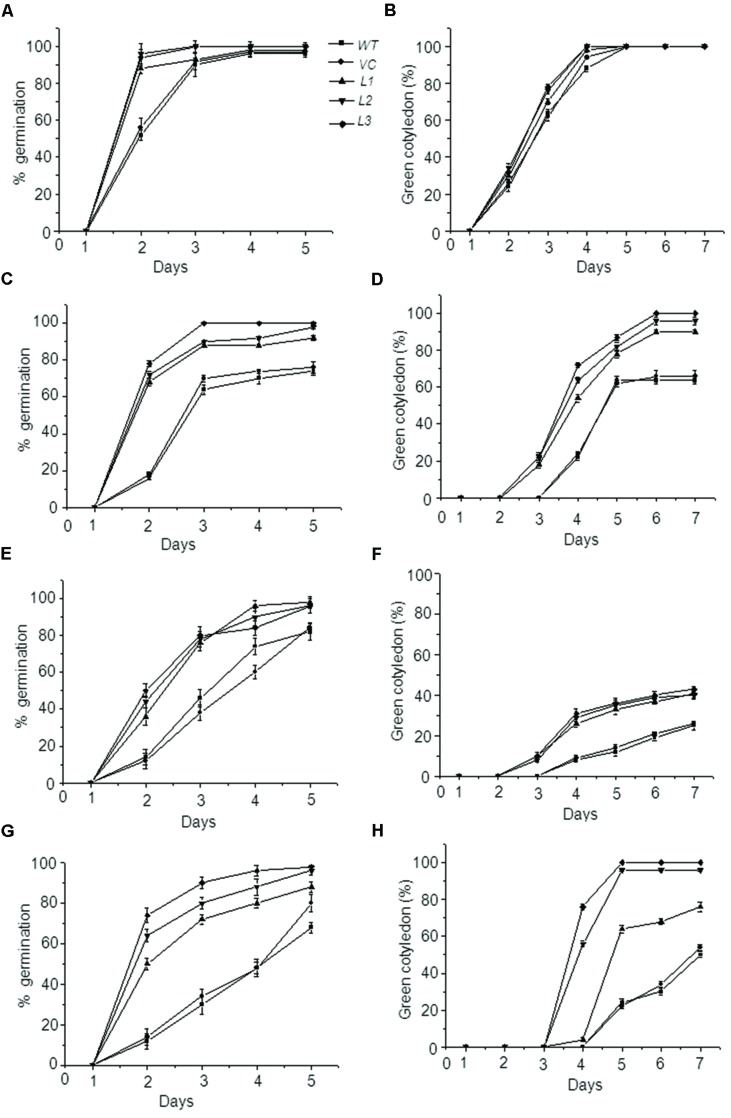
**Germination and seedling establishment of *OsHSP18.2* overexpressing seeds under abiotic stresses.** Germination percentage **(A,C,E,G)** and green cotyledon emergence **(B,D,F,H)** of WT, VC, and transgenic seeds overexpressing *OsHSP18.2* scored under **(A,B)** Control conditions, **(C,D)** Heat (45°C), **(E,F)** Salt (150 mM NaCl), and **(G,H)** Dehydration (-0.4 MPa PEG). Age matched seeds were surface sterilized and plated on either ½ MS or ½ MS supplemented for various stresses. Plates were stratified at 4°C for 3 days and transferred to growth chamber at 22 ± 2°C. Values are mean ± SE of three independent sets (*n* = 50).

Germination performance of these transgenic seeds under various stress situations were also evaluated, since seed vigor also implies the ability to complete germination under widely variable environmental conditions. Germination of WT, VC, and transgenic seeds was assessed under heat, dehydration, and salt stress and was considered complete when radicle emerged beyond testa. While moderate level of these stresses (i.e., 37°C heat, 100 mM NaCl and -0.25 MPa PEG) did not much affect the germination (data not shown) but slightly elevated stresses revealed a significant difference in germination pattern among control and overexpressing seeds (**Figure 5**). In normal conditions, *OsHSP18.2* transformed and control seeds showed 100% germination. For heat stress, seeds were treated at 45°C for 1 h and then plated on ½ MS, results revealed that >90% of transgenic seeds could complete germination in all lines by the end of 5 days whereas only 70% seeds of WT and VC could germinate by this time (**Figure 5C**). At 150 mM NaCl concentration, >90% seeds of transgenic lines could germinate compared to 80% of WT and VC (**Figure 5E**). Dehydration stress was provided by -0.4 MPa PEG and as expected overexpression lines performed better than control lines (**Figure 5G**). All seeds were monitored for 7 days and seedling establishment was checked which was taken as the emergence of green cotyledons over this period as indicated in previous study (Silva-Correia et al., 2014). Results suggest that in addition to seed germination a significant difference was also observed for seedling establishment among control and *OsHSP18.2* overexpressing seeds in adverse conditions (**Figure 5**).

Overall results conclude with strong evidence that *OsHSP18.2* is an aging responsive protein which plays an important role in maintaining seed vigor and longevity as well as seedling emergence by protecting structural damage to proteins and restricting ROS accumulation during prolonged storage.

## Discussion

Research on small HSPs has been more emphasized toward plant stress tolerance, however, their role in seed physiology has not been elucidated properly, particularly in rice where majority of the sHSPs are specifically expressed in seed (Sarkar et al., 2009). In this study, our proteomic analysis identified OsHSP18.2 as an aging responsive protein whose abundance significantly increases after artificial aging, indicating its involvement in rice seed vigor and longevity. Previous studies clearly revealed that similar molecular events accompany both in artificial and natural seed aging and thus artificial aging induced protein is also likely to be induced by natural aging (Catusse et al., 2008; Rajjou et al., 2008). Subsequently, we showed that *OsHSP18.2* transcript is markedly increased at the late maturation stage and is highly abundant in dry seeds and sharply decreases after germination. This expression pattern of *OsHSP18.2* also supports its involvement in desiccation tolerance and longevity as other protective molecules that are generally associated with seed desiccation tolerance and longevity in orthodox seeds are also accumulated in maturation phase and are highly abundant in dry seeds. High transcript accumulations of various sHSPs during seed maturation and in dry seed were also reported in several plant species like pea, sunflower, *Arabidopsis* and rice (Coca et al., 1994; DeRocher and Vierling, 1994; Wehmeyer et al., 1996; Sarkar et al., 2009; Omar et al., 2011; Zhou et al., 2012). Accelerated aging induced increase in *OsHSP18.2* transcript level in rice seed also strengthens our hypothesis that OsHSP18.2 indeed participates in seed vigor and longevity. Notably, a high abundance of sHSPs was reported in beech seeds stored for 8 years (Kalemba and Pukacka, 2008). In addition, studies also suggest that induction of certain sHSPs in embryo is a routine part of seed development and seed desiccation tolerance program (DeRocher and Vierling, 1994).

Moreover, analysis of *OsHSP18.2* promoter also revealed motifs like 2SSEEDPROTBANAPA, CANBNNAPA, DPBFCOREDCDC3, EBOXBNNAPA, NAPINMOTIFBN, and SEF4MOTIFGM7S which are responsible for seed and embryo specific expression (**Table 1**). In addition to this there are many abiotic stress and hormone responsive elements as well which include the heat stress response element HSE (GAANNTTCNNGAA) and well known dehydration responsive element CBFHV (RYCGAC), MYBCORE and MYCCONSENSUSAT thus explaining a higher expression of the transcript during heat and dehydration stress. Surprisingly, few low temperature responsive sequences were also present but our real time experiments revealed not much induction of OsHSP18.2 transcript during cold stress. DPBFCOREDCDC3 (ACACNNG) and DRE2COREZMRAB17 (ACCGAC) responsible for ABA inducible expression are also present. Expression data available on GENEVESTIGATOR also reveals slightly up regulation in response to ABA and Salicylic acid treatment (Hruz et al., 2008). Multiple repeats of pollen specific expression element POLLEN1LELAT52 (AGAAA) present in the promoter suggests that developmentally regulated expression of sHSPs during desiccation associated plant stages such as pollen formation, sporulation, and seed development emphasizes their crucial role during the acquisition of desiccation tolerance (DeRocher and Vierling, 1994). Interestingly, some highly conserved sequence motifs like UPRMOTIFIAT (CCACGTCA) and UPRMOTIFIIAT (CCNNNNNNNNNNNNCCACG) for unfolded protein response are also present as these elements have been also shown to occur in the promoters of other heat shock protein (HSP90) in *Arabidopsis*. Thus, these sequences might drive the expression of OsHSP18.2 gene during the accumulation of unfolded polypeptides in the cell.

Subsequently, our biochemical study clearly demonstrated that OsHSP18.2 forms oligomeric complex, is dodecameric in nature and function as a molecular chaperone (**Figure 3**). Studies on wheat sHSP16.9 also demonstrated a dodecameric structure of this heat shock protein although homologs from other species have been shown to have structures with subunits ranging between 2 and >48 subunits (van Montfort et al., 2001; Basha et al., 2012). This is well known that sHSPs function in plant stress adaptation by binding to vulnerable cellular proteins during stress conditions, prevent their aggregation and thus hold them in a competent state for refolding by other chaperones (Ehrnsperger et al., 1997; Veinger et al., 1998; Murakami et al., 2004). Our data clearly establishes that OsHSP18.2 works as a chaperone at high temperature and prevents irreversible thermal inactivation of CS. Comparative studies have shown that addition of pea HSP18.1 significantly increased the refolding of Luciferase even though DnaK system was alone sufficient for stabilization and refolding thus emphasizing its role in restricting denaturation and assisting refolding (Lee and Vierling, 2000). Taken together the increased accumulation of *OsHSP18.2* in later stages of seed maturation and aging and its function as a molecular chaperones, suggests that OsHSP18.2 might protect vulnerable cellular proteins during maturation drying, desiccation and aging in seeds. This hypothesis fits well as during seed maturation, desiccation, and storage, seed, particularly embryo faces severe dehydration and oxidative stress that can potentially damage seed proteins (Tamarit et al., 1998; McDonald, 1999; Ingrosso et al., 2000; Rajjou and Debeaujon, 2008). In addition, *OsHSP18.2* was shown to be highly induced in rice seedlings exposed to thermal stress, indicating its participation also in thermal stress tolerance.

Our subsequent functional studies essentially demonstrated that *OsHSP18.2* has the ability to improve seed vigor and longevity (**Figure 4**). This improved seed vigor and longevity correlates well with the reduced deleterious ROS accumulation in transformed seeds. Similar to our observation, heterologous expression of a sHSP from sacred lotus in *Arabidopsis* also lead to enhanced germination of transgenic seeds after accelerated aging treatment (CDT; Zhou et al., 2012). In addition, certain members of the sHSP family have been known to be involved in scavenging of these reactive oxygen species (Härndahl et al., 1999; Fedoroff, 2006). Our results also demonstrated faster and better germination and seedling establishment under adverse situations in *OsHSP18.2* overexpressing lines. Collectively, our findings clearly establish that OsHSP18.2 is seed vigor and aging responsive protein which functions as a molecular chaperone in stabilizing the cellular proteins from irreversible damage and containing ROS accumulation during seed development particularly during seed maturation, drying and storage. Finally, our results also propose that this gene can be a good candidate to improve seed vigor and longevity in terms of better seed germination and seedling establishment in wide environmental stress conditions.

## Conflict of Interest Statement

The authors declare that the research was conducted in the absence of any commercial or financial relationships that could be construed as a potential conflict of interest.

## Abbreviations

ABA: abscisic acid
ACD: α-crystallin domain
CDT: controlled deterioration treatment
CS: citrate synthase
DAB: 3,3′-diaminobenzidine tetrahydrochloride
DTNB: 5,5′-dithiobis (2-nitrobenzoic acid)
PEG: poly ethylene glycol
sHSP: small heat shock Protein
TZ: 2,3,5-triphenyltetrazolium chloride
VC: vector control
WT: wild type
