# Supplementary material for: Differentially expressed seed aging responsive heat shock protein OsHSP18.2 implicates in seed vigor, longevity and improves germination and seedling establishment under abiotic stress
Source: Front Plant Sci. 2015 Sep 14;6:713. doi: 10.3389/fpls.2015.00713 (PMC4568394; doi:10.3389/fpls.2015.00713)
Supplement: Supplementary file 1 [file Image_1.PDF]

Supplementary Material

Differentially expressed seed aging responsive heat shock protein OsHSP18.2 implicates in seed vigor, longevity and improves germination and seedling establishment under abiotic stress.

Harmeet Kaur, Bhanu Prakash Petla, Nitin Uttam Kamble, Ajeet Singh, Venkateswara Rao, Prafull Salvi, Shraboni Ghosh and Manoj Majee*

National Institute of Plant Genome Research, New Delhi, India.

***Correspondence:** Dr. Manoj Majee, National Institute of Plant Genome Research, Aruna Asaf Ali Marg, New Delhi- 110067, India.

manojmajee@nipgr.ac.in

1 Supplementary Figures and Tables

1.1 Supplementary Figures

A

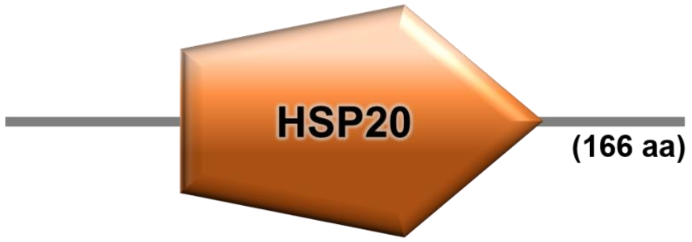

B

1 MESAMFGLETPLMTALQHLLDIPDGEGGAAGKQGATGGPTRAYVRDARAMAATPADVKDL 60

61 PGAYAFVVDMPGLKSSDIKVQVEEERLLVISGERRRGGGEEEKEESCKYLRMERRMGKFM 120

121 RKFVLPDNADVDKISAVCQDGVLTVTVEKLPPPEPKKPKTIEVKVA 166

Supplementary Figure 1. Domain structure of OsHSP18.2. (A) Pictorial description of the α-crystalline domain (ACD) characteristic of HSP20 family, (B) ACD (56 – 149aa) highlighted in the amino acid sequence of OsHSP18.2.

```
OsHSP18.2          1 MESAMFGLETPLMTALQHLLDIPDGEGGAAGKQGATGGPTRAYVRDARAM     50
                     |:...|    |:::.|:.:|::|:..    ..:.....|:|.|:|||:||
AtHSP17.0          1 MDLGRF----PIISILEDMLEVPEDH----NNEKTRNNPSRVYMRDAKAM     42

OsHSP18.2         51 AATPADVKDLPGAYAFVVDMPGLKSSDIKVQVEEERLLVISGERRRGGGE    100
                     |||||||.:.|.||||||||||:|..:||||||.:.:||:||||:|   |
AtHSP17.0         43 AATPADVIEHPNAYAFVVDMPGIKGDEIKVQVENDNVLVVSGERQR---E     89

OsHSP18.2        101 EEKEESCKYLRMERRMGKFMRKFVLPDNADVDKISAVCQDGVLTVTVEKL    150
                     .::.|..||:|||||||||||||.||:|||:|||||||.||||.|||:||
AtHSP17.0         90 NKENEGVKYVRMERRMGKFMRKFQLPENADLDKISAVCHDGVLKVTVQKL    139

OsHSP18.2        151 PPPEPKKPKTIEVKVA    166
                     |||||||||||:|:||
AtHSP17.0        140 PPPEPKKPKTIQVQVA    155
```

Supplementary Figure 2. Sequence alignment of OsHSP18.2 and its homologue in Arabidopsis, AtHSP17.0. Sequences were aligned with the EMBOSS Needle pairwise sequence alignment tool and share 59% identity at amino acid level (Tair ID - AT5G12020.1).

1.2 Supplementary Tables

Supplementary Table 1. Primers used in this study.

MM1	F	CATATGATGGAGAGCGCCATGTTCGGG	OsHSP18.2 Forward primer for bacterial expression
MM2	R	CTCGAGCGCGACCTTGACCTCGATGGTC	OsHSP18.2 Reverse primer for bacterial expression
MM3	F	GAAGCTTATGGAGAGCGCCATGTTCGGG	OsHSP18.2 Forward primer for plant expression
MM4	R	GTCTAGATCATCACGCGACCTTGACCTCG	OsHSP18.2 Reverse primer for plant expression
MM5	F	GAGAAGGAGGAGTCGTGCAAGT	OsHSP18.2 Forward primer for real time PCR
MM6	R	CACACGGCGGAGATCTTGT	OsHSP18.2 Reverse primer for real time PCR
MM7	F	CCTCATGAAGATCCTGACGG	OsActin Forward primer for real time PCR
MM8	R	TCATGTCCCTCACAATTTCCC	OsActin Reverse primer for real time PCR
